# Training on an inexpensive tablet-based device is equally effective as on a standard laparoscopic box trainer: A randomized controlled trial: Erratum

**DOI:** 10.1097/MD.0000000000006216

**Published:** 2017-02-17

In the article “Training on an inexpensive tablet-based device is equally effective as on a standard laparoscopic box trainer: A randomized controlled trial”,^[1]^ Figures 1 and 2 have been incorrectly matched to their legends. The image in Figure 1 actually represents a standard box trainer, and the image in Figure 2 represents a tablet-based cardboard box trainer.
